# Genetic and Phenotypic Characterization of the Etiological Agent of Canine Orchiepididymitis Smooth *Brucella* sp. BCCN84.3

**DOI:** 10.3389/fvets.2019.00175

**Published:** 2019-06-07

**Authors:** Caterina Guzmán-Verri, Marcela Suárez-Esquivel, Nazareth Ruíz-Villalobos, Michel S. Zygmunt, Mathieu Gonnet, Elena Campos, Eunice Víquez-Ruiz, Carlos Chacón-Díaz, Beatriz Aragón-Aranda, Raquel Conde-Álvarez, Ignacio Moriyón, José María Blasco, Pilar M. Muñoz, Kate S. Baker, Nicholas R. Thomson, Axel Cloeckaert, Edgardo Moreno

**Affiliations:** ^1^Programa de Investigación en Enfermedades Tropicales (PIET), Escuela de Medicina Veterinaria, Universidad Nacional, Heredia, Costa Rica; ^2^Facultad de Microbiología, Centro de Investigación en Enfermedades Tropicales, Universidad de Costa Rica, San José, Costa Rica; ^3^ISP, INRA, Université François Rabelais de Tours, Nouzilly, France; ^4^Centro Nacional de Referencia en Bacteriología, Instituto Costarricense de Investigación y Enseñanza en Nutrición y Salud (INCIENSA), Cartago, Costa Rica; ^5^IDISNA and Departamento de Microbiología y Parasitología, Instituto de Salud Tropical, Universidad de Navarra, Pamplona, Spain; ^6^Unidad de Producción y Sanidad Animal, Instituto Agroalimentario de Aragón-IA2, CITA-Universidad de Zaragoza, Zaragoza, Spain; ^7^Pathogen Genomics, Wellcome Trust Sanger Institute, Hinxton, United Kingdom; ^8^Institute for Integrative Biology, University of Liverpool, Liverpool, United Kingdom

**Keywords:** *Brucella*, *Brucella melitensis*, *Brucella suis*, *Brucella canis*, brucellosis, dog, species, epididymitis

## Abstract

Members of the genus *Brucella* cluster in two phylogenetic groups: classical and non-classical species. The former group is composed of *Brucella* species that cause disease in mammals, including humans. A *Brucella* species, labeled as *Brucella* sp. BCCN84.3, was isolated from the testes of a Saint Bernard dog suffering orchiepididymitis, in Costa Rica. Following standard microbiological methods, the bacterium was first defined as “*Brucella melitensis* biovar 2.” Further molecular typing, identified the strain as an atypical “*Brucella suis*.” Distinctive *Brucella* sp. BCCN84.3 markers, absent in other *Brucella* species and strains, were revealed by fatty acid methyl ester analysis, high resolution melting PCR and *omp25* and *omp2a/omp2b* gene diversity. Analysis of multiple loci variable number of tandem repeats and whole genome sequencing demonstrated that this isolate was different from the currently described *Brucella* species. The smooth *Brucella* sp. BCCN84.3 clusters together with the classical *Brucella* clade and displays all the genes required for virulence. *Brucella* sp. BCCN84.3 is a *species nova* taxonomical entity displaying pathogenicity; therefore, relevant for differential diagnoses in the context of brucellosis. Considering the debate on the *Brucella* species concept, there is a need to describe the extant taxonomical entities of these pathogens in order to understand the dispersion and evolution.

## Introduction

The *Brucella* genus comprises two phylogenetically related clusters: classical and non-classical (1). The former cluster is a compact group composed of *Brucella melitensis, Brucella abortus, Brucella suis, Brucella canis, Brucella neotomae, Brucella ceti, Brucella pinnipedialis, Brucella ovis, Brucella microti, Brucella papionis*, and *Brucella* sp. F5/99. All these species infect and produce disease in mammals, displaying host preference. Members of this cluster are non-motile, devoid of plasmids and their genomes show nucleotide identities of >99% (1, 2). The first six *Brucella* species of this cluster are zoonotic and can infect humans (3–5).

Non-classical *Brucella* species, also known as the “BO clade,” cluster in a discrete group that includes the fast-growing *Brucella inopinata* and BO2 strains isolated in humans as well as *Brucella* species living in frogs (1). *Brucella vulpis*, isolated from red foxes in Australia, is more distant to BO clade and contains unique genetic information related to soil bacteria not encoded in classical *Brucella* organisms (1). Bacteria of the BO clade and *B. vulpis* display nucleotide identities of 97–98% with those of the classical clade. The species of this cluster also share genes with the soil bacteria *Ochrobactrum* spp. and show key sequence differences in central genes such as 16S rRNA and *recA*, as distinctive features (1). With the sole exception of *B. inopinata*, these *Brucella* species possess an O-chain lipopolysaccharide (LPS) structure that departs from that of the classical *Brucella* species (1, 6). This feature hampers the straightforward recognition of non-classical *Brucella* infections in animals.

Identification of the classical *Brucella* species and strains by traditional bacteriological and molecular methods is not straightforward. This is due to the high phenotypic and genotypic resemblance among different members of the genus (3, 7). For this reason, many *Brucella* strains isolated from various animal species have been misclassified or not fully characterized (8, 9). One clear example of clinical relevance has been the discovery of *B. neotomae* as a human pathogen, which was wrongly classified as an atypical *B. abortus* strain by classical bacteriological methods (10). With the advent of sophisticated molecular tools and whole genome sequence analysis (WGSA), the correct identification of *Brucella* species was achieved (1, 4).

Here, we describe the phenotypic and genotypic properties of a new classical pathogenic smooth *Brucella* sp., isolated from a Saint Bernard dog suffering orchiepididymitis. After its primary isolation in 1984 in Costa Rica (11), the strain was first assigned as an atypical strain of *B. melitensis* biovar 2 (12).

## Materials and Methods

### Clinical Case and Bacterial Isolation

A 4-year male domestic Saint Bernard dog from the Central Valley of Costa Rica showing testicular lesions, was brought to the Hospital of the Veterinary School of the National University, in 1984. After hospitalization, the owner was informed of all procedures and clinical studies and gave her written consent. All protocols and actions undertaken to diagnose the disease were under the Veterinary Hospital guidance established in 1980. The protocols used in 1984, were those approved by the “Ley General de Salud” N° 5395, and “Disposiciones sobre Matrícula y Vacunación de Perros” N° 2391.

After anamnesis and clinical examination, the dog was subjected to surgery and both testes removed. Rose Bengal test (13) was used to determine the presence of antibodies against smooth *Brucella*. Histopathological examination of the testes was performed following previous protocols (14). For bacterial isolation, blood and testicular samples were cultured in blood-agar plates. The plates were incubated at 37°C under the presence or the absence of 10% CO_2_ atmosphere. The bacterial colonies were identified as *Brucella* sp. at the Bacteriology Laboratory of INCIENSA, Costa Rica (11). The isolate (code *Brucella* sp. BCCN84.3) was freeze-dried and submitted for further bacteriological and molecular typing, as described below.

### Bacterial Phenotypic Characterization

The *Brucella* sp. BCCN84.3 was subjected to classical bacteriological typing (Table 1) following established protocols (13). Reference *Brucella* strains were used for comparative purposes (Supplementary Table 1). Total lipids were extracted and analyzed as described elsewhere (15) and resolved on silica gel 60 high-performance TLC plates (Merck Chemicals) using n-propanol/propionic acid/chloroform/water (3:2:2:1) and developed by charring with 15% (v/v) sulfuric acid in ethanol (16). Processing of the fatty acid methyl ester for taxonomical identification and dendrogram assembly were carried out as described before (17). Extraction of LPS by SDS-proteinase K protocol was performed as described previously (18). LPS was analyzed in 12 or 15% polyacrylamide gels and stained by the periodate-alkaline silver method (19). An immune serum obtained from *B. melitensis* 16M infected rabbits (20), either plain or absorbed with cells from rough Per mutant strain derived from *B. abortus* 2308W, was used for assessing anti-smooth-LPS reactivity. Immune serum obtained from *B. abortus* Per immunized rabbit (21) was used for anti-rough-LPS reactivity. Western blots and ELISA with a collection of monoclonal antibodies (Mabs) for the detection of specific *Brucella* surface antigens were performed as described elsewhere (22, 23). Susceptibility to polymyxin B was determined by estimating the minimal inhibitory concentration on Müller-Hinton agar (Becton Dickinson, Izasa), following the e-test (Liofilchem, Werfen) method (24).

**Table 1:** Microbiological characterization of *Brucella* sp. BCCN84.3 and comparison with *Brucella* reference strains.

**Strains**	**RTD phage lysis^a^**				**CO_**2**_ requirement**	**Urease**	**Serum agglutination against^b^**		**Growth on dyes, μg/mL^c^**				
	**Tb**	**Wb**	**Iz**	**R/C**			**A**	**M**	**Thionin**		**Basic fuchsin**		**O safranin**
									**10**	**20**	**10**	**20**	**100**
*Brucella* sp. BCCN84.3^d^	–	+	+	–	No	+	+	–	+	+	+	+^c^	+
*B. abortus* 2308W	+	+	+	–	No	+	+	–	–	–	+	+	+
*B. suis* 1330	–	+	+	–	No	+	+	–	+	+	–	–	–
*B. melitensis* 16M	–	–	+	–	No	+	–	+	+	+	+	+	+
*B. ovis 63/290*	–	–	–	+	Yes	+	–	–	+	–	–	–	–
*B. canis* CR12	–	–	–	+	No	+	–	–	+	–	–	–	–
*B. neotomae* 5K/33	+	–	+	+	No	+	+	–	–	–	–	–	
*B. microti* CCM 4915	–	+			No	+	–	+	+	+	+	+	
*B. ceti*, B1/94	–	+	–	–	No	+	+	–	+	+	+	+	+
*B. pinnipedialis* B2/94	–	–	+	–	Yes	+	+	–	+	+	+	+	+

a *RTD, routine test dilution of phages Tbilisi (Tb), Weybridge (Wb), Izatnagar (Iz), and rough type Wb derivative (R/C)*.

b *Serum against LPS epitopes, measured as agglutination with monospecific serum*.

c *Dye concentrations expressed in μg/mL of culture medium and plates incubated under 10% CO_2_ atmosphere*.

d *The strain was oxidase positive and readily produced H_2_S*.

### Genotypic and Phylogenetic Characterization

Bacterial DNA was extracted with DNeasy Blood & Tissue kit from QIAGEN or Promega Wizard Genomic DNA Purification kit as per manufacturer's instructions. DNA was stored at −70°C until used. Bruce-ladder v2.0 PCR for the differentiation of *Brucella* species and strains was carried following previous protocols (25). Suis-ladder PCR assay for *B. suis* biovar typing and the discrimination of *B. suis* and *B. canis* was performed as described before (26).

Two different real-time PCRs, for the detection of *Brucella* genus and *B. suis* were performed as previously described (27). Additionally, two different high-resolution melting PCR assays (HRM-PCR) for the specific detection and discrimination of *B. canis* and *B. melitensis* were performed following previous protocols (27), using a DNA concentration of 1.5 ng/μL and a Type-it HRM-PCR Kit (QIAGEN) in a reaction volume of 25 μL with a Rotor-Gene Q (QIAGEN). Control DNAs from *B. canis* RM 6/66, *B. melitensis* 16M, *B. suis* 1330 and *B. neotomae* 5K/33 were extracted with DNeasy Blood & Tissue kit from QIAGEN, and stored at −80°C until used. The conditions were one cycle at 50°C for 2 min and one cycle at 95°C for 10 min, followed by 40 cycles at 95°C for 5 s and a cycle at 60°C for 30 s, with data acquired at 60°C in the green channel. After amplification, an HRM-PCR was performed when needed from 73 to 88°C at a rate of 0.03°C per step.

Multiple loci variable number of tandem repeats analysis (MLVA16) and the corresponding cladograms were generated according to described protocols (17, 28) using the MLVA-NET database (29). Values obtained for each MLVA16 marker are in Supplementary Data Sheet 1.

WGSA was performed at the Wellcome Trust Sanger Institute on Illumina platforms according to in-house protocols (30, 31). For WGSA assembly and alignment sequencing reads were *de novo* assembled using a Velvet Optimiser (32). In order to overcome possible genome deviation through serial cultivation, the strain deposited in 1984 in the *Brucella* Culture Collection Nouzilly (BCCN) was also sequenced and deposited at DDBJ/ENA/GenBank under the accession NQLX00000000; Accession *Brucella* sp. BCCN84.3 (NQLX00000000; BioSample SAMN07488835). WGSA from representative *Brucella* strains used for comparative purposes were obtained from GenBank (Supplementary Data Sheet 1). Low length and N50 scaffold sequences were not included in the analysis. Automatic annotation of the assembly was performed with the Prokka program (33). Genome sequence data was deposited at the European Nucleotide Archive under accession code ERS568777 and at DDBJ/ENA/GenBank under the accession NQLX00000000; BioSample SAMN07488835 (Supplementary Data Sheet 1). The 9 and 21 loci schemes of Multi Locus Sequence typing (MLST) were performed *in silico* by BLAST comparison with a set of specific primers (34) and the assembled scaffolds as input. The results were confirmed by querying the matched sequences or “amplicons” at the Brucella MLST Database (https://pubmlst.org/brucella/) (Supplementary Data Sheet 1).

### Phylogenetic Reconstruction

Two *Ochrobactrum* species and *Brucella* isolates were used for phylogenetic reconstruction (Supplementary Data Sheet 1). The 25 WGSA were aligned by *bwa* and mapped with SMALT v.0.7.4 against *B. suis* 1330, with an average mapping of 89.41% when excluding *Ochrobactrum*. Single Nucleotide Polymorphisms (SNPs) were called using SAMtools (35), and 451213 variable sites were extracted using SNP sites (36). The general features of all 25 assemblies annotated by Prokka were used to perform a pangenome analysis (36). Both SNPs and core genome alignments were individually used to each produce a maximum likelihood phylogenetic reconstruction with RAxML v8.2 (37). The phylogenetic trees were rooted using *Ochrobactrum anthropi* ATCC49188 and *Ochrobactrum intermedium* LMG3301.

### A Specific Search for Regions of Interest

Regions of interest were searched through *bwa* alignment and SMALT mapping, or BLAST comparison against *B. canis* ATCC RM6/66 (NC_010103.1-NC_010104.1), *B. suis* 1330 (NC_004310.3-NC_004311.2), *B. abortus* 9-941 (NC_006932.1-NC_006933.1), *B. abortus* 2308W (ERS568782), *B. melitensis* 16M (NC_003317.1-NC_003318.1), and *B. microti* CCM 4915 (NC_013119.1-NC_013118.1). The number of SNPs, insertions and deletions in each gene were recorded. BLAST comparisons between *Brucella* sp. BCCN84.3, *B. canis* RM6/66 and *B. suis* 1330 were performed and visualized with the Artemis Comparison Tool (38). The presence of recombination events was analyzed by Genealogies Unbiased By recomBinations In Nucleotide Sequences (39) and visualized by Phandango (40). Southern blot analysis was performed as described previously (41) using the IS elements IS*711* and ISBme1 as probes on EcoR1-digested DNA.

For phylogenetic reconstruction, comparisons among *omp2a* (BAW_10633) and *omp2b* (BAW_10634) porin gene sequences were assessed through multiple sequence alignments. Characterization of Omp2a and omp2b have been used as molecular tools for the description of *Brucella* species since 2007 (42), Sanger sequence data from 14 classical *Brucella* strains were visualized, edited, aligned, and analyzed in MEGA version 7 (43). The resulting alignment of 1,223 positions was used to build a phylogenetic tree by the maximum likelihood method based on the Tamura-Nei model (44). The tree with the highest log likelihood was selected. All the positions containing gaps or missing data were eliminated. Initial trees for the heuristic search were obtained automatically by applying the Neighbor-Join and BioNJ algorithms to a matrix of pairwise distances estimated using the maximum composite likelihood approach and then by selecting the topology with a superior log-likelihood value.

## Results

The anamnesis revealed that the Saint Bernard dog was imported from the United States as a puppy to Costa Rica, in 1980. The animal lived in the city of Heredia, Costa Rica and was never in contact with farm animals or mated. Upon arrival to the Veterinary Medicine School, the dog showed unwillingness to walk, general lethargy, refusal to eat, aspermia, fever, enlargement of the scrotum and testicles with local dermatitis and scrotal pain. The animal did not show any rashes, abdominal pain, visceral enlargement or local adenopathy. Platelets and leukocyte counts were normal. Pathological inspection showed bilateral enlargement of the epididymis and inflammation as well mild necrosis of both testes. Histopathological examination of testicular tissue revealed necrotizing foci and granulomatous inflammation. Since the serum of the animal showed positive agglutination in Rose Bengal test for brucellosis, it was not necessary to perform any other serological tests. The presumptive clinical diagnosis was orchiepididymitis due to brucellosis.

Serological diagnosis was confirmed by isolation of smooth *Brucella* sp. from testicular tissue after 1 week of culture in blood agar. The dog was treated orally with doxycycline (20 mg/Kg), three times a day for 14 days. Then streptomycin (11 mg/Kg) was administrated intramuscular every 12 h during 14 days. After treatment the dog showed improvement; however, the animal was not followed afterward.

**Figure 1:**
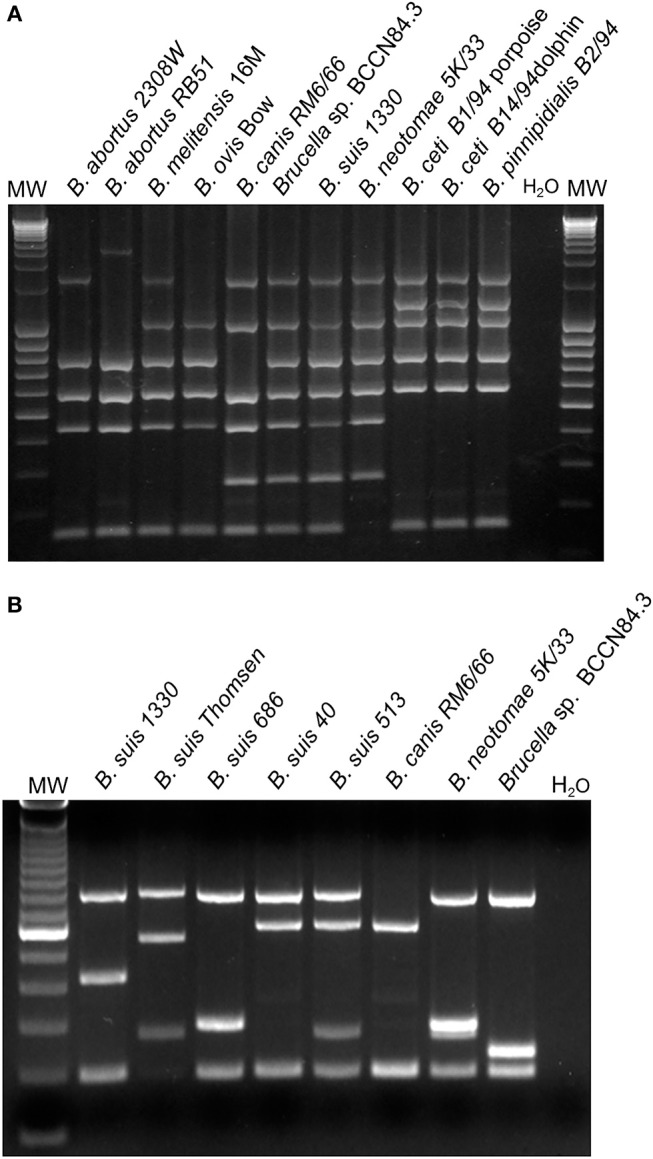
Typification of *Brucella* sp. BCCN84.3 by Bruce-ladder and Suis-Ladder. **(A)** Bruce-ladder analysis does not distinguish between *B. suis* species and BCCN84.3. **(B)** Suis-Ladder identified a characteristic pattern for *Brucella* sp. BCCN84.3, different from those of *B. suis* and *B. canis*.

Since the isolate displayed an atypical bacteriological profile (11), the strain was sent to the Station de Pathologie de la Reproduction, INRA, Centre de Tours-Nouzilly, France, for typing. The strain presented an atypical oxidative metabolic profile with particularly high levels for L-glutamic acid and L-asparagine utilization. The strain was coded as *Brucella* sp. BCCN84.3 and identified as an atypical *B. melitensis* biovar 2 (12). Moreover, Bruce-ladder did not distinguish between *B. suis* biotype 1 and *Brucella* sp. BCCN84.3 (Figure 1A). However, the *Brucella* sp. BCCN84.3 strain displayed a different Suis-ladder profile departing from *B. suis* and *B. canis* strains (Figure 1B).

**Figure 2:**
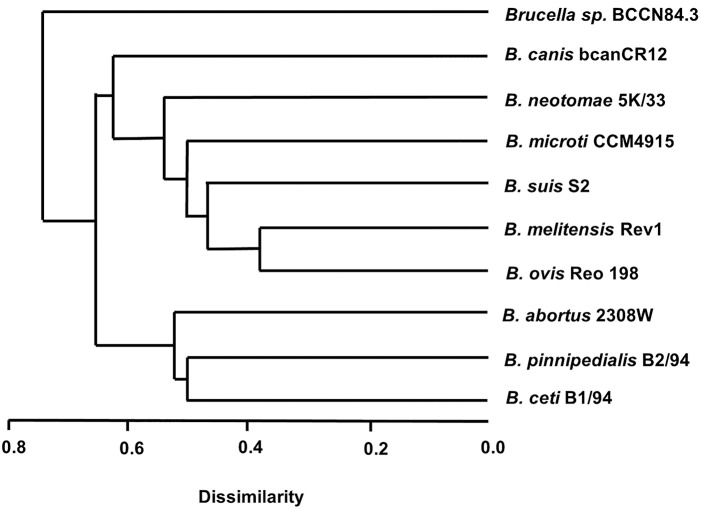
Dendrogram of the fatty acid methyl esters of different *Brucella* extracts. Notice that the *Brucella* sp. BCCN84.3 stands alone in relation to the classical species. For values of retention times of fatty acid methyl esters (see Supplementary File 2).

**Figure 3:**
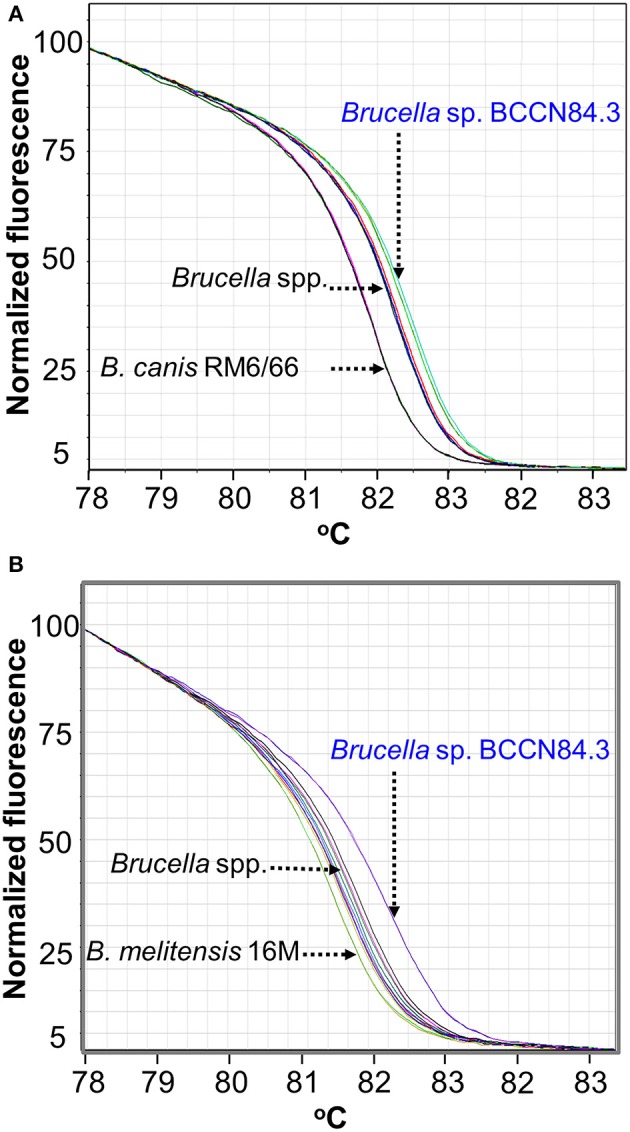
HRM-PCR analysis of *Brucella* sp. BCCN84.3. **(A)** HRM-PCR assay using primers designed for *B. canis* **(A)** or *B. melitensis* **(B)** (27) clearly show that *Brucella* sp. BCNN84.3 has an HRM profile different from other classical *Brucella* species.

Conventional phenotyping did not allow ascription to any of the currently accepted *Brucella* nominal species (Tables 1, 2). However, the *Brucella* sp. BCCN84.3 fatty acid methyl esters profile suggested a different taxonomical rank (Figure 2). Likewise, plus-minus real-time PCR analysis using DNA from *Brucella* sp. BCCN84.3 was positive for the *Brucella* genus and *B. suis*. HRM-PCR analysis using specific primers for *B. canis* (Figure 3A) or *B. melitensis* (Figure 3B) showed that the profile of the BCCN84.3 strain was unique as compared to classical *Brucella* species.

**Table 2:** Cell envelope characteristics of *Brucella* sp. BCCN84.3 and comparison with reference *Brucella* strains.

	**Major lipids**		**Reactivity with serum to**		**Resistance to cationic peptides (PmxB MIC μg/ml)**
	**Phospholipids**	**Aminolipids**	**O-chain**	**R-LPS**	
*Brucella* sp. BCCN84.3	PC; PE; PG; CL	OL	A>M	+	12
*B. canis* CR12	PC; PE; PG; CL	OL	–	+	12
*B. microti* CCM 4915	PC; PE; PG; CL	OH-OL; OL	A>M	–	>16
*B. melitensis* 16M	PC; PE; PG; CL	OL	M>A	+	16
*B. suis* 1330	PC; PE; PG; CL	OL	A>M	+	16
*B. abortus* 2308W	PC; PE; PG; CL	OL	A>M	+	4

*PC, phosphatidylcholine; PE, phosphatidylethanolamine; PG, phosphatidylglicerol; CL, cardiolipine, OL, ornithine lipids; OH-OL, hydroxylated ornithine lipids: PmxB, polymixin B*.

**Figure 4:**
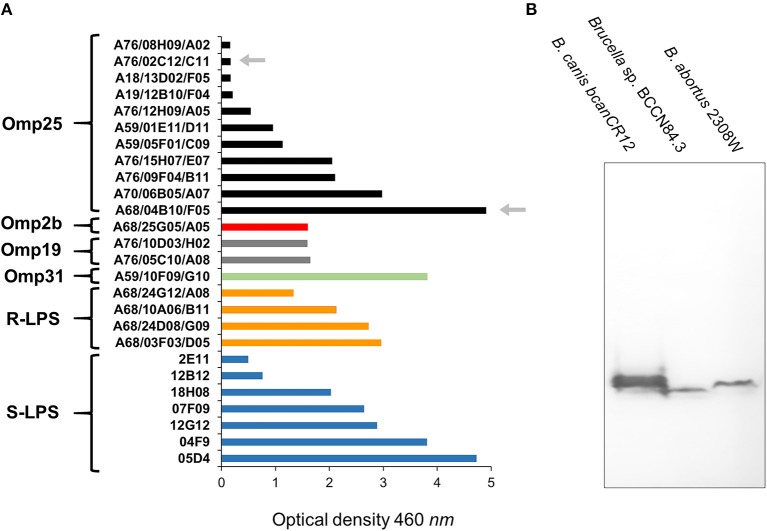
The binding intensity of a collection of Mabs against *Brucella* sp. BCCN84.3. **(A)** A collection Mabs against *Brucella* Omp31, Omp35, Omp2b, Omp19, smooth LPS (S-LPS), and rough-LPS (R-LPS) were tested by ELISA against sonicated *Brucella* sp. BCCN84.3 sonicated cells and the binding compared with *B. abortus* or *B. melitensis* cells. The arrows indicate differential reactivity of both Mabs against a conformational epitope of the Omp25 in comparison to *B. abortus* or *B. melitensis*. For details of the ELISA assay see Cloeckaert et al. (22) **(B)** WB with Mabs 68/04B10/F05 against Omp25, identifies a slightly lower molecular weight protein in *Brucella* sp. BCCN84.3 strain in relation to other classical *Brucella* species.

Following previous experiments (22, 45, 46), no significant differences in bindings against *Brucella* sp. BCCN84.3 rough-LPS, smooth-LPS, Omp2b, Omp19, and Omp31 were detected by ELISA (Figure 4A). In contrast, when compared with other brucellae (46), a distinct profile against the *Brucella* sp. BCCN84.3 Omp25 was attained (Figure 4A). Mab A68/04B10/F05 against the Omp25 conformational epitope reacted with *Brucella* sp. BCCN84.3, the Mab A76/02C12/C11 (also directed against a conformational epitope, 43) reaction was negative. A slightly lower molecular weight of the Omp25 was identified in the *Brucella* sp. BCCN84.3, as compared to the *B. canis* and *B. abortus* counterparts (Figure 4B). This pattern agrees with the length of *omp25* (BAW_10696 locus), which is slightly shorter than other *omp25* genes of classical *Brucella* species.

**Figure 5:**
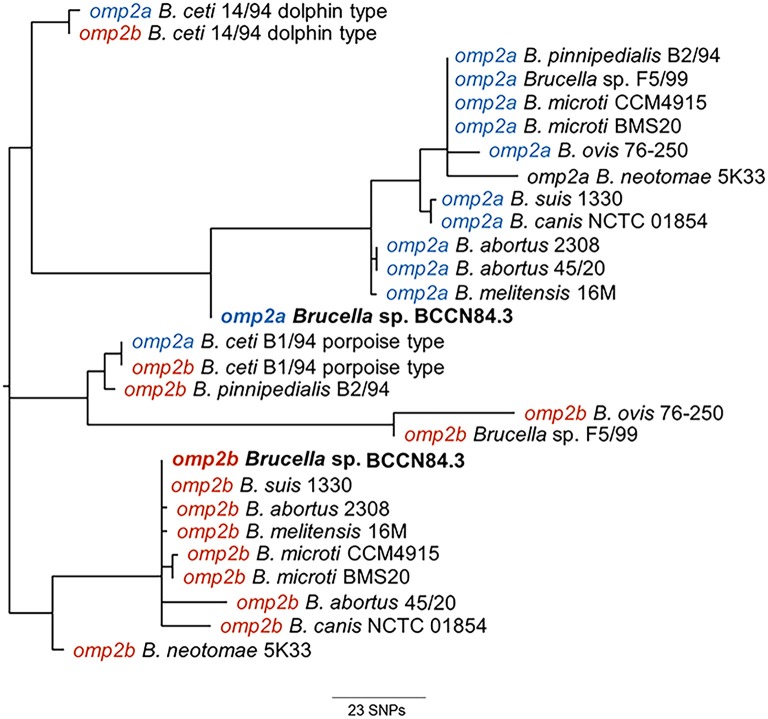
Phylogenetic tree from CLUSTAL Wallis aligned *omp2a* and *omp2b* nucleotide sequences of BCCN84.3 and other *Brucella* strains. The analysis reveals the separation in BCCN84.3 *omp2a* and *omp2b* clusters. The *Brucella* sp. BCCN84.3 omp2a is somewhat closer to the omp2b cluster; probably due to a recombination event in the 5′region, which is identical to the latter porin sequence.

Phylogenetic analysis of the *Brucella* sp. BCCN84.3 porin sequences showed a separation in the *omp2a* and *omp2b* corresponding clusters (Figure 5). However, the *Brucella* sp. BCCN84.3 *omp2a* was somewhat closer to the *omp2b* cluster, due to a putative recombination event in a region close to the 5′, which is identical to the porin sequence of the latter (47).

**Figure 6:**
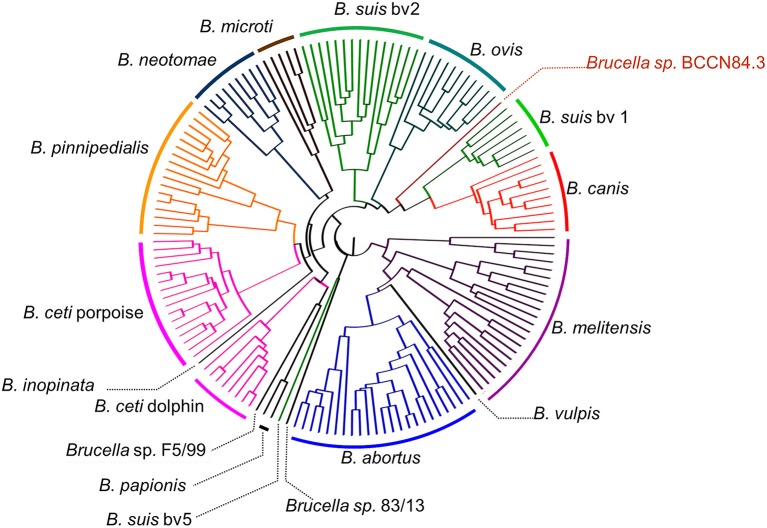
Dendrogram based on MLVA16 analysis of *Brucella* species and strains representatives. The *Brucella* sp. BCCN84.3 showed a MLVA16 profile different from that of the classical smooth *Brucella* species; consistent with a previous report using a different MLVA strategy (48). MLVA-NET for *Brucella*. MLVA web service, CNRS. http://microbesgenotyping.i2bc.paris-saclay.fr/ (accessed 21 December, 2017).

The *Brucella* sp. BCCN84.3 formed a distinct branch in relation to other species, as revealed by the MLVA16 analysis (Figure 6). This result is in agreement with a previous analysis, using a somewhat different MLVA strategy (48). WGSA demonstrated that the overall genomic structure of the *Brucella* sp. BCCN84.3 isolate corresponds to a new species of classical brucellae, with a size of 3.26 Mb. Parallel sequencing of the strain conserved in the BCCN collection (named *Brucella* sp. BCCN84.3) confirmed the stability of the genome. When both WGS were compared, no deletions or insertions were found between the strains and, only three SNPs were detected at intergenic regions.

**Figure 7:**
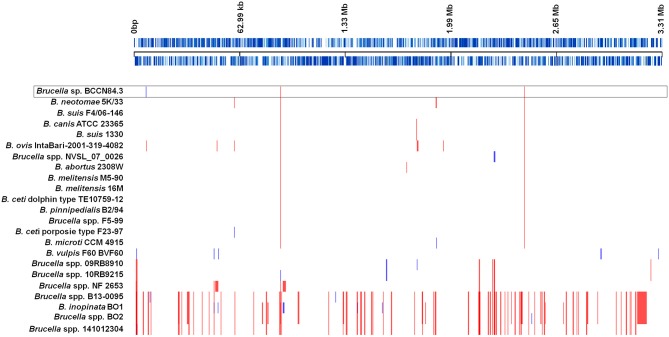
Recombination events in representative *Brucella* species. Each event is shown by a vertical block ordered along the genome. Upper black line represents the approximate coordinates in base pairs according to the reference *B. suis* 1330; each blue line represents a coding sequence in the reference. Red blocks are recombination events shared by more than two genomes included; blue blocks are unique. Classic *Brucella* species show few recombination regions; however, a higher number was detected in the non-classical clades. The *Brucella* sp. BCCN84.3 is highlighted by a box.

**Figure 8:**
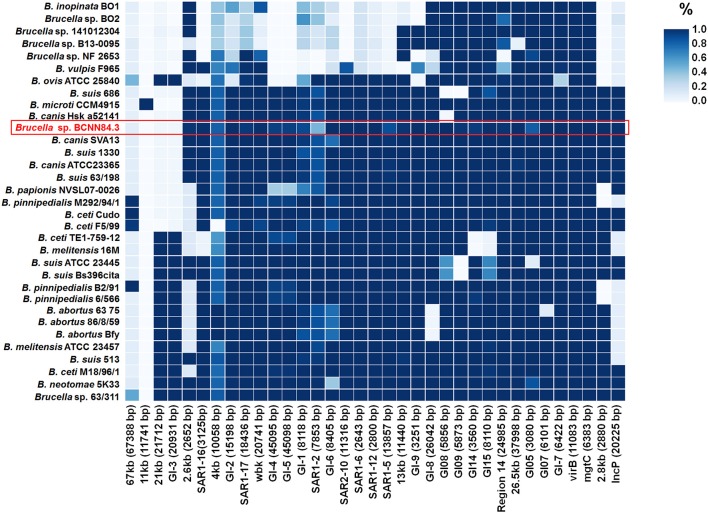
The presence and absence of anomalous regions or genomic islands in the *Brucella* genomes. The upper right color scale represents the percentage of the island present in each genome, where the darker blue color means that the whole region is present. *Brucella* sp. BCCN84.3 is highlighted by a red box.

As other classical brucellae, *Brucella* sp. BCCN84.3 presents two chromosomes with no plasmids, no major recent recombination events (Figure 7) and a similar number of anomalous regions (Figure 8). The genes encoding for virulence factors such as smooth type LPS, VirB operon, Bac, cyclic glucans, flagellum-like, and BvrR/BvrS system are conserved (Supplementary Data Sheet 1). The *B. canis* genomic island GI_FeGSH_ coding for iron uptake enzymes and parts of the glutathione pathway (49) is not present in the *Brucella* sp. BCCN84.3. Putative genes in loci BAW_10265 coding for the TIR domain-containing protein BtpA claimed to be a VirB effector of the type IV secretion system and to modulate microtube dynamics (50), and for putative integrases (BAW_10237; BAW_10274) are also absent. The *manBOAg* (BAW_10538) putatively involved in the synthesis of mannose of the LPS core (51) was 48 bp shorter than the *B. melitensis* (BMEI1396) and about the same size as *B. ovis* (BOV_0540) and *B. abortus* 2308W (BAW_10538) counterparts. The number of *IS711* elements identified by southern blot ranged from 6 to 7. Due to the repetitive nature of the IS elements, determination of the exact number by WGSA on Illumina platforms was not possible.

**Figure 9:**
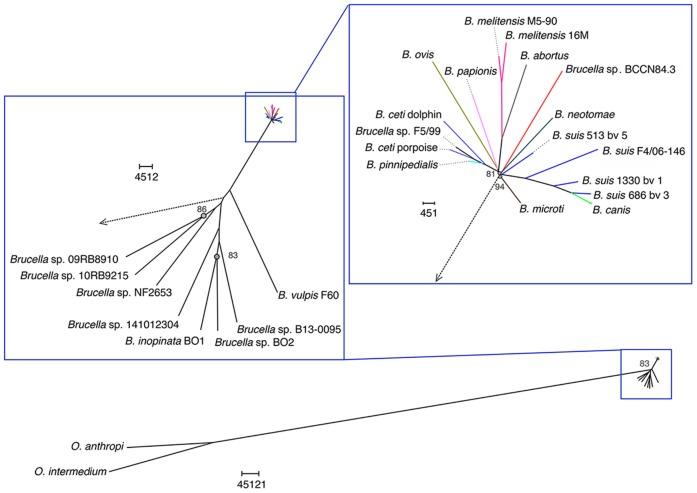
Phylogenetic relationship of *Brucella* sp. BCCN84.3 with other *Brucella* species and *Ochrobactrum* sp. *Brucella* BCCN84.3 results are colored in red. Branching points with bootstrap values lower than 100 are indicated by a gray dot and small gray font next to it. Segments of the tree were magnified by the use of Dendroscope version 3.5.8 in order to increase resolution; the adapted scale is indicated next to each magnified region. A blue square highlights all *Brucella* species; the classic species are within the upright square, which includes the *Brucella* sp. BCCN84.3.

A total of 205,055 SNPs were found among the *Brucella* genomes (Supplementary Data Sheet 1) and were used for phylogenetic analysis using *O. anthropi* and *O. intermedium* cluster as an outgroup. The general topology of the SNPs based tree was consistent with previous studies (1). *Brucella* sp. BCCN84.3 showed 7,281 polymorphic sites as compared to *B. suis* 1330, of those 5,911 were located in coding regions with a dN/dS ratio of 0.54 (*p*-value = 0.00). This shows a compact cluster harboring classical *Brucella* species and a more dispersed clade harboring the BO group (Figure 9). Within the classical cluster, *Brucella* sp. BCCN84.3 branches alone (Figure 9). This branching order does not fully agree with the classical MLVA16 dispersion. *In silico* identical matches of the 9 loci included in the MLST-9 profile were not able to classify the *B. abortus* sp. BCCN84.3 into a sequence type. The-21 loci MLST profile did not provide more information, 20 loci showed identical match, except for the *ddlA* locus, that partially matched to the allele 26, so no further typing was achieved by this scheme.

## Discussion

Canine brucellosis, caused by *B. canis*, is difficult to diagnose by serological assays due to the extensive cross-reaction of antigens with smooth brucellae (52, 53). The unambiguous diagnosis of *B. canis* infections is just carried out after the isolation and identification of the bacterium (14) or molecular typing (26, 54). In contrast, when positive serological reactions against smooth brucellae arise in dogs presenting clinical signs of brucellosis, the presumptive diagnosis seems straightforward and commonly attributed to *B. melitensis, B. abortus* or *B. suis* (55–59). However, a detailed identification of the smooth *Brucella* strains isolated from dogs is seldom performed.

We were unable to trace the source of the Saint Bernard dog infection. The dog was imported as a puppy from the United States to Costa Rica. Whether the infection remained latent or it was acquired *de novo* in Costa Rica, is unknown. This is not trivial since there are several reports describing “atypical *B. suis* strains” isolated from dogs in different countries, including in the United States. For instance, in the same year as the *Brucella* sp. BCCN84.3 was isolated, a collection of “atypical *B. suis*” strains, which were also unusually resistant to fuchsin, were described in various countries (60). A new *B. suis* biovar was suggested for these atypical strains, some of them isolated from dogs and humans. Likewise, in the same year, an “atypical *B. suis* biotype 1” was also isolated in Brazil, from the testes of a dog suffering orchitis (61). In a survey carried out in 674 dogs in Georgia, United States, it was established that nine dogs presented positive serological reactions against smooth *Brucella* antigens (58). *Brucella* organisms were isolated from the canine testes displaying necrotizing, suppurative epididymitis and orchitis. After conventional biochemical assays and 16SrRNA sequencing, the bacterial strains were assigned to the “*B. suis*” group. Unfortunately, these latter isolates were destroyed, precluding any further detailed characterization. More recently, several dogs were reported to be infected with “*B. suis”* in Australia; even though not all dogs were in contact with wild boars (62). In all these studies the bacterial strains were identified by conventional methods or rRNA PCR analysis; though, none of these methods are capable to unambiguously discern among the various *Brucella* classical species (63). The initial bacteriological characterization of the *Brucella* sp. BCCN84.3 was also misleading. It was only after genomic analyses that it became clear that the strain belonged to a new taxonomic entity.

From the genomic perspective, the *Brucella* sp. BCCN84.3 is a new taxonomical entity, since it departs phylogenetically from other strains, being the closest relative *B. neotomae* but distinct from this species. The total number of SNPs between *Brucella* sp. BCCN84.3 and *B. suis* 1330 (7281 SNPs) is bigger than the number between *B. suis* and *B. abortus* (6790 SNPs), two well-recognized species. It is also closer to the number that separates *B. ovis* st. IntaBari-2001-319-4082 from *B. suis* st. 1330 (7499 SNPs). Considering the zoonotic potential of *Brucella* species, a correct identification by molecular methods is becoming mandatory. Moreover, in the light of distinct host preferences (64) and differences in WGSA (1), the various *B. suis* strains need to be taxonomically reevaluated, since they seem to represent a collection of different *Brucella* species. In particular *B. suis* biovar 5 isolated from rodents which branches closer to *B. microti* (4) and the two clusters composed, on one hand by *B. suis* biovars 2 and 3, and on the other hand by *B. suis* biovars 1 and 4 (4). The problem with this latter cluster is the close phylogenetic relationship of *B. canis* with *B. suis* biovar 4 (4), which requires an idiosyncratic solution. The correct classification of *Brucella* species is particularly relevant in countries like Costa Rica, in which *B. melitensis* and *B. suis* are absent (65), or in countries in which bovine, caprine, and swine brucellosis have been eradicated from livestock, but that still have pathogenic *Brucella* infecting wildlife (66). In this regard, the differential diagnosis of the various *Brucella* species and strains is a requirement for taking the infection source.

*Brucella* sp. BCCN84.3 is a *species nova*. More isolates of this bacterium are necessary and additional epidemiological and biological information needs to be collected before assigning the corresponding taxonomical species name. In spite of this, and taking into account the difficulties surrounding the debate on the *Brucella* species concept (5, 7), it is mandatory to describe the extant taxonomical entities in order to understand the dispersion and evolution of these important pathogens.

The fact that *Brucella* sp. BCCN84.3. has the ability to invade the reproductive tract of dogs, may favor the venereal transmission of this bacterium, as it the case of *B. canis* which rapidly disperse in kennel facilities. We do not know the zoonotic potential of *Brucella* sp. BCCN84.3. However, it is a smooth strain that possesses all the virulent machinery for being pathogenic for humans and other animals. Moreover, the fact that it was isolated from a domestic dog increases the zoonotic risk.

## Data Availability

Publicly available datasets were analyzed in this study. This data can be found here: https://www.ebi.ac.uk/ena/data/view/ERS568777.

## Ethics Statement

This is a clinical case. The dog was brought by its owner for therapy. Following the regular arrangements for hospitalization, the owner was informed for all procedures and clinical studies and gave her written consent. All protocols and actions undertaken to diagnose the disease were under the Veterinary Hospital guidance established in 1980. The protocols used in 1984, were those approved by the Ley General de Salud N° 5395, and Disposiciones sobre Matrícula y Vacunación de Perros N° 2391.

## Author Contributions

EM, CG-V, and AC conceived the study. EM, CG-V, IM, NT, and JB obtained funding. EC and EM performed the isolation of the bacterium. CC-D performed fatty acid analysis. CG-V, MS-E, KB, AC, NR-V, MZ, EV-R, and MG performed genomics analyses. RC-Á, BA-A, and IM performed the LPS and lipid characterization. JB, EC, PM, and IM performed the bacteriological analysis. EM, CG-V, MS-E, AC, NR-V, NT, MG, and CC-D performed data interpretation. EM and CG-V wrote the paper. All authors read and approved the manuscript content.

### Conflict of Interest Statement

The authors declare that the research was conducted in the absence of any commercial or financial relationships that could be construed as a potential conflict of interest.

## Supplementary Material

Supplementary Table S1 Control and reference *Brucella* species and strains used for typing.

Supplementary Data Sheet 1 (i) Excel files displaying the *Brucella* strains used in MLVA16; (ii) the *Brucella* sp. BCCN84.3 WGSA statistics, the metadata of WGSA included in the phylogenetic reconstruction; (iii) the SNPs summary in comparison to *B. suis* 1330 as reference, (iv) the mutations found in *Brucella* sp. BCCN84.3 in genes related to virulence as compared to the indicated locus/gene name; (v) the genomic islands the comprises metadata of WGSA included in the analysis and genomic islands reference information; (vi) the metadata of sequences included in the phylogenetic reconstruction using omp2a and omp2b genes, and; (vii) the retention time of the fatty acid methyl ester to construct dendrogram of Figure 2.
