# Corrigendum: Genetic and Phenotypic Characterization of the Etiological Agent of Canine Orchiepididymitis Smooth *Brucella* sp. BCCN84.3

**DOI:** 10.3389/fvets.2019.00253

**Published:** 2019-08-02

**Authors:** Caterina Guzmán-Verri, Marcela Suárez-Esquivel, Nazareth Ruíz-Villalobos, Michel S. Zygmunt, Mathieu Gonnet, Elena Campos, Eunice Víquez-Ruiz, Carlos Chacón-Díaz, Beatriz Aragón-Aranda, Raquel Conde-Álvarez, Ignacio Moriyón, José María Blasco, Pilar M. Muñoz, Kate S. Baker, Nicholas R. Thomson, Axel Cloeckaert, Edgardo Moreno

**Affiliations:** ^1^Programa de Investigación en Enfermedades Tropicales (PIET), Escuela de Medicina Veterinaria, Universidad Nacional, Heredia, Costa Rica; ^2^Facultad de Microbiología, Centro de Investigación en Enfermedades Tropicales, Universidad de Costa Rica, San José, Costa Rica; ^3^ISP, INRA, Université François Rabelais de Tours, Nouzilly, France; ^4^Centro Nacional de Referencia en Bacteriología, Instituto Costarricense de Investigación y Enseñanza en Nutrición y Salud (INCIENSA), Cartago, Costa Rica; ^5^IDISNA and Departamento de Microbiología y Parasitología, Instituto de Salud Tropical, Universidad de Navarra, Pamplona, Spain; ^6^Unidad de Producción y Sanidad Animal, Instituto Agroalimentario de Aragón-IA2, CITA-Universidad de Zaragoza, Zaragoza, Spain; ^7^Pathogen Genomics, Wellcome Trust Sanger Institute, Hinxton, United Kingdom; ^8^Institute for Integrative Biology, University of Liverpool, Liverpool, United Kingdom

**Keywords:** *Brucella*, *Brucella melitensis*, *Brucella suis*, *Brucella canis*, brucellosis, dog, species, epididymitis

In the original article, there was an error. In the Funding statement is written that MZ was granted with a fellowship from SEP, Universidad de Costa Rica. The correct Initials are MS-E.

A correction has been made to the **Funding** statement:

This work was supported by FEES-CONARE, Costa Rica; Fondo Institucional de Desarrollo Académico (FIDA), Universidad Nacional; Wellcome Trust; CITA-INIA, Spain (project Bru-Epidia 291815-FP7/ERANET/ANIHWA); MINECO (AGL2014-58795-CA), and Aragon Government (Consolidated Group A14). Authors from the Sanger Institute were supported by Wellcome Trust (098051). NR-V was partially sponsored by a scholarship from the University of Costa Rica. KB was founded by a Wellcome Trust Postdoctoral Training Fellowship for Clinicians (106690/Z/14/Z). MS-E was granted with a fellowship from SEP, Universidad de Costa Rica.

Additionally, an inaccurate sentence was included in Author Contributions stating that MZ kept the bacterium in ISP, INRA collection.

A correction has been made to **Author Contributions** statement:

EM, CG-V, and AC conceived the study. EM, CG-V, IM, NT, and JB obtained funding. EC and EM performed the isolation of the bacterium. CC-D performed fatty acid analysis. CG-V, MS-E, KB, AC, NR-V, MZ, EV-R, and MG performed genomics analyses. RC-Á, BA-A, and IM performed the LPS and lipid characterization. JB, EC, PM, and IM performed the bacteriological analysis. EM, CG-V, MS-E, AC, NR-V, NT, MG, and CC-D performed data interpretation. EM and CG-V wrote the paper. All authors read and approved the manuscript content.

The authors apologize for these errors and state that they do not change the scientific conclusions of the article in any way. The original article has been updated.

