# Supplementary material for: Genetic and Phenotypic Characterization of the Etiological Agent of Canine Orchiepididymitis Smooth Brucella sp. BCCN84.3
Source: Front Vet Sci. 2019 Jun 7;6:175. doi: 10.3389/fvets.2019.00175 (PMC6568212; doi:10.3389/fvets.2019.00175)
Supplement: Supplementary Table S1 — Control and reference Brucella species and strains used for typing. [file Table_1.pdf]

Supplementary Table S1. Control and reference *Brucella* species and strains used for typing

**Species and strains**	**Host specie**	**Common name**	**Reference**
Brucella abortus 2308W	*Bos taurus*	Cow	(Suárez-Esquivel et al., 2016)
Brucella suis 1330	*Sus scrofa*	domestic pig	(López-Goñi et al., 2008)
Brucella melitensis 16M	*Capra aegagrus*	Goat	(López-Goñi et al., 2008)
Brucella ovis Reo 198	*Ovis aries*	Ram	(López-Goñi et al., 2008)
Brucella ovis 8/290 (BOW)	*Ovis aries*	Ram	(López-Goñi et al., 2008)
Brucella canis bcanCR12	*Canis familiaris*	Domestic dog	(Chacón-Díaz et al., 2015)
Brucella canis RM6/66	*Canis familiaris*	Domestic dog	(López-Goñi et al., 2011)
Brucella neotomae 5K/33	*Neotoma lepida*	Wood rat	(Suárez-Esquivel et al., 2017)
Brucella microti CCM4915	*Microtus arvalis*	Common vole	(López-Goñi et al., 2011)
Brucella ceti B1/94	*Phocoena phocoena*	Porpoise	(Hernández-Mora et al., 2008)
Brucella ceti B14/94	*Delphinus delphis*	Common dolphin	(Hernández-Mora et al., 2008)
Brucella pinnipedialis B2/94	*Phoca vitulina*	Common seal	(Hernández-Mora et al., 2008)
Brucella suis Thomsen	*Sus scrofa*	domestic pig	(López-Goñi et al., 2011)
Brucella suis 686	*Sus scrofa*	domestic pig	(López-Goñi et al., 2011)
Brucella suis 40	*Sus scrofa*	domestic pig	(López-Goñi et al., 2011)
Brucella suis 513	*Sus scrofa*	domestic pig	(López-Goñi et al., 2011)
Brucella suis S2	*Sus scrofa*	domestic pig	(Bosseray and Plommet 1990)

References

Bosseray, N., Plommet, M. (1990). Brucella suis S2, Brucella melitensis Rev. 1 and Brucella abortus S19 living vaccines: residual virulence and immunity induced against three Brucella species challenge strains in mice. Vaccine. 8,462-568.

Chacón-Díaz, C., Altamirano-Silva, P., González-Espinoza, G., Medina, M.C., Alfaro-Alarcón, A., Bouza-Mora, L., Jiménez-Rojas, C., Wong, M., Barquero-Calvo, E., Rojas, N., Guzmán-Verri, C., Moreno, E., Chaves-Olarte, E. (2015). Brucella canis is an intracellular pathogen that induces lower proinflammatory response than smooth zoonotic counterparts. Infect. Immun. 83,4861-4870.

Hernández-Mora, G., González-Barrientos, R., Morales, J.A., Chaves-Olarte, E., Guzmán-Verri, C., Barquero-Calvo, E., De-Miguel, M.J., Marín, C.M., Blasco, J.M., Moreno, E. (2008). Neurobrucellosis in stranded dolphins, Costa Rica. Emerging Infect. Dis. 14,1430-1433.

López-Goñi, I., García-Yoldi, D., Marín, C.M., de Miguel, M.J., Barquero-Calvo, E., Guzmán-Verri, C., Albert, D., Garin-Bastuji, B. (2011). New bruce-ladder multiplex PCR assay for the biovar typing of Brucella suis and the discrimination of Brucella suis and Brucella canis. Vet. Microbiol. 154,152-155.

López-Goñi, I., García-Yoldi, D., Marín, C.M., de Miguel, M.J., Muñoz, P.M., Blasco, J.M., Jacques, I., Grayon, M., Cloeckaert, A., Ferreira, A.C., Cardoso, R, Corrêa de Sá, M.I., Walravens, K., Albert, D., Garin-Bastuji, B. (2008). Evaluation of a multiplex PCR assay (Bruce-ladder) or molecular typing of all *Brucella* species, including the vaccine strains. J. Clin. Microbiol. 46,3484-3487.

Suárez-Esquivel, M., Ruiz-Villalobos, N., Castillo-Zeledón., A, Jiménez-Rojas, C., Roop, R.M., Comerci, D.J., Barquero-Calvo, E., Chacón-Díaz, C., Caswell, C.C., Baker, K.S., Chaves-Olarte, E., Thomson, N.R., Moreno, E., Letesson, J.J., De Bolle, X., Guzmán-Verri, C. (2016). *Brucella abortus* strain 2308 Wisconsin genome: importance of the definition of reference strains. Front. Microbiol. 7,1557.

Suárez-Esquivel, M., Ruiz-Villalobos, N., Jiménez-Rojas, C., Barquero-Calvo, E., Chacón-Díaz, C., Víquez-Ruiz, E., Rojas-Campos, N., Baker, K.S., Oviedo-Sánchez, G., Amuy, E., Chaves-Olarte, E., Thomson, N.R., Moreno, E., Guzmán-Verri, C. (2017). Brucella neotomae infection in humans, Costa Rica. J. Emerg. Infect. Dis. 23,997-1000.
